# A‐kinase‐interacting protein 1 promotes EMT and metastasis via PI3K/Akt/IKKβ pathway in cervical cancer

**DOI:** 10.1002/cbf.3547

**Published:** 2020-05-13

**Authors:** Xiujuan Zhang, Shuxian Liu, Yongqing Zhu

**Affiliations:** ^1^ Department of Ultrasound Fujian Medical University Union Hospital Fuzhou China; ^2^ Department of Biomedical Sciences Fudan University Shanghai China; ^3^ Department of Gynecology Obstetrics and Gynecology Hospital of Fudan University Shanghai China

**Keywords:** A‐kinase‐interacting protein 1, cervical cancer, epithelial‐mesenchymal transition, NF‐κB pathway

## Abstract

Overexpression of A‐kinase‐interacting protein 1 (AKIP1) has been reported in prostate and breast cancers. Nevertheless, the clinical potential of AKIP1 during the development of cervical cancer (CC) remains unclear. A series of experiments involving BdU, colony formation, wound healing and cell invasion assays were performed to determine cell proliferation, migration and invasion, respectively. Gene expression changes were validated by qRT‐PCR, Western blotting and immunocytochemistry. We found that AKIP1 expression is increased in CC cell lines and tissue specimens from CC patients. The elevated AKIP1 expression in primary tumours was related to lymph node metastasis in CC patients. In addition, we observed that overexpression of AKIP1 promotes CC cell proliferation. Enhanced expression of AKIP1 facilitated the migration and invasion of CC cells by inducing NF‐κB‐dependent epithelial‐mesenchymal transition (EMT). Moreover, mechanistic investigations revealed that AKIP1 induced nuclear translocation and phosphorylation of the p65 NF‐κB subunit through the PI3K/Akt/IKKβ pathway. Conversely, enhanced expression of phosphatase and tensin homologue (PTEN) inhibited this signalling pathway and restored an epithelial phenotype to CC cells in the presence of overexpressed AKIP1. Our results indicate that AKIP1 promotes the EMT and metastasis in CC by activating NF‐κB signalling through the PI3K/Akt/IKKβ pathway, suggesting that AKIP1 could be a pivotal regulator of an EMT axis in CC.

**Significance of the study:**

AKIP1 expression in the samples of CC patients and in in vitro tumour cell lines provides evidence that expression of AKIP1 in CC contributes to cancer progression. Our findings indicate that AKIP1 promotes the epithelial‐mesenchymal transition and metastasis in CC by activating NF‐κB signalling through the PI3K/Akt/IKKβ pathway, suggesting that AKIP1 is a pivotal regulator of an EMT axis in CC. AKIP1 could be implicated as a potential therapeutic target as well as a valuable biomarker for evaluating prognosis for patients with CC.

## 1 INTRODUCTION

Cervical cancer (CC) ranks the fourth in terms of prevalence and mortality among women worldwide.^1^ The presence of retroperitoneal lymph node metastasis is a key to predict the prognosis of CC at an early‐stage and requires a combined treatment of cisplatin‐based chemotherapy and adjuvant pelvic radiation.^2^ Nevertheless, no current clinical model can accurately predict the behaviour of potentially metastatic tumours either before or after surgery. Therefore, the monitoring and treatment of metastatic tumours of CC remain a great challenge.

Model of metastasis can be established by cell migration, invasion and the process, epithelial‐mesenchymal transition (EMT). EMT was initially found to promote the escape and migration of cells.^3^ In addition, it is critical to the metastasis of CC.^4^, ^5^

A‐kinase‐interacting protein 1 (AKIP1) is generated from a library that contained mRNA of cells from matched normal and breast tumour tissues.^6^ Previous studies show that AKIP1 can be retained inside the nuclei through interaction with nuclear factor‐κB (NF‐κB), thus activating the NF‐κB‐dependent transcription.^7^, ^8^ Elevated AKIP1 expression occurs in both tumour cells and in the plasma of patients diagnosed with many types of cancers.^9^ The clinical potential of AKIP1 in CC development remains unclear. Furthermore, activated NF‐κB is involved in CC progression through regulating the process of EMT.^10^, ^11^, ^12^

This study investigated the clinical importance and biological functions of AKIP1 in CC. Furthermore, we tested the hypothesis that AKIP1 can induce EMT in CC by activating NF‐κB–associated pathway.

## 2 MATERIAL AND METHODS

### 2.1 Cells and reagents

CC cell lines (SiHa [ATCC Cat# HTB‐35, RRID: CVCL_0032], C33A [ATCC Cat# CRM‐HTB‐31, RRID: CVCL_1094], HeLa [ATCC Cat# CRL‐7923, RRID: CVCL_0030], and CaSki [ATCC Cat# CRL‐1550, RRID: CVCL_1100]), as well as human cervical epithelial cells, were purchased from ATCC. Cell culture was performed with Dulbecco's modified eagle medium containing 10% fetal bovine serum (FBS) (Thermo Fisher, Waltham, MA, USA), streptomycin (100 μg/mL) and penicillin (100 U/mL) at 37°C under 5% CO_2_. PI3K inhibitor LY294002, IKK inhibitor Bay117082, and AKIP1 protein were purchased from Sigma‐Aldrich (St. Louis, MO).

### 2.2 Tissue specimens

The study received approval from the Ethics Committee of Obstetrics and Gynaecology Hospital of Fudan University. All patients were diagnosed pathologically and the stage of the tumour was classified according to World Health Organization (WHO) criteria. All patients signed the forms of consent. Specimens were collected during tumour resection of 57 randomly selected CC patients treated with radical hysterectomy and lymphadenectomy at Obstetrics and Gynaecology Hospital of Fudan University. The inclusion criteria were (a) pathologically confirmed diagnosis of CC, (b) accepting curative resection, defined as the complete removal of the neoplasm macroscopically, and (c) complete and detailed clinic pathological data. The exclusion criteria were (a) having received preoperative cancer treatment or (b) showing evidence of other malignancies.

### 2.3 Successful establishment of CC cell lines that stably express AKIP1

Lentiviral vectors carrying the AKIP1 gene and vectors encoding a scrambled RNA were purchased (GeneChem, Shanghai, China). CaSki cells and HeLa cells were infected with these lentiviral vectors. Polyclonal cells expressing positive signals were subsequently chosen by a fluorescence‐activated cell sorting (FACS) flow cytometer for the following procedures. Total RNA was isolated from the cells and the level of AKIP1 expression was measured through reverse transcriptase quantitative PCR (qRT‐PCR).

### 2.4 Stable suppression of AKIP1 in CC cells

With a scrambled small hairpin RNA (shRNA) lentiviral vector as control, cell lines CaSki cells and HeLa cells were transfected with a lentiviral vector expressing shRNA against AKIP1 (GeneChem, Shanghai, China), their total RNA and protein were extracted. qRT‐PCR and western blotting were performed to quantify AKIP1 RNA and protein, respectively.

### 2.5 Determination of cell proliferation

This procedure was carried out with the Cell‐Light EdU Apollo488 in‐vitro Imaging Kit (Cat: C10338‐1, RiboBio, Guangzhou, China) and repeated three times. In brief, cell equilibration was performed for 2 hours in 10 μM EdU, and fixed in 4% paraformaldehyde. Sequentially, after permeabilization in Triton X‐100 (0.3%), cells were stained in EdU, during which the nuclei were stained by 4′, 6‐diamidino‐2‐phenylindole (5 μg/mL). Under the microscope, number of EdU‐positive cells were determined in five randomly selected fields (×100), and the average was taken.

### 2.6 Colony formation

Cells were inoculated at a density of 200 cells/well in 6‐well plate in duplicate, at 37°C for 14 days, and thereafter, adherent cells were rinsed using phosphate buffered saline (PBS) twice. Subsequently, cells were stained by haematoxylin. Colonies containing >50 cells were selected to determine the colony formation efficiency.

### 2.7 Transfection

Cells seeded at 5 × 10^5^/well in a 6‐well plate in media supplemented with 10% FBS to reach 50% confluency 12 hours before transfection, followed by transfection with AKIP1 siRNA (50 nmol/L), the negative control (NC) (Guangzhou RiboBio Co., Guangzhou, China), or 5 μg AKIP1 plasmids (Shanghai GeneChem Co., Shanghai, China) using transfection reagent Lipofectamine 2000 (Invitrogen, Carlsbad, CA), and collected for subsequent experiments after 48 to 72 hours.

### 2.8 RNA isolation and qRT‐PCR

Easy Pure RNA Kit (Cat: ER201‐01, Transgen Biotech Co., Ltd. Beijing, China) was used to isolate the total RNA and cDNA was synthesized for qRT‐PCR with GAPDH as internal reference. Relative mRNA expression was calculated by the 2^−δδCT^ method. This procedure was repeated three times. The primers used were as follows: AKIP1, 5′‐CCA ACC CTT AGT GCT TCC‐3′ for the forward and 5′‐TCGACTCGCCTCTGTGATA‐3′ for the reverse; GAPDH, 5′‐CAGCCTCAAGATCATCAGCA‐3′ for the forward and 5′‐TGTGGTCATGAGTCCTTCCA‐3′ for the reverse.

### 2.9 Western blot analysis

Proteins (30 μg) were loaded on a precast gel in equal amounts to perform electrophoresis. After the electrophoresis, proteins on the gel were transferred to a nitrocellulose membrane which was blocked by incubating for 1 hour with 5% milk at room temperature. Primary antibodies for fibronectin(Cat: 26836), N‐cadherin(Cat: 13116), E‐cadherin(Cat: 14472), p‐p65 (Ser536)(Cat: 3033), α‐Tubulin(Cat:2125), Lamin A/C(Cat:4777), and β‐Actin(Cat: 4970) p‐IKKβ (Ser177)(Cat:2078), IKKβ(Cat:8943), p‐IκBα (Ser32)(Cat:5210), and IκBα(Cat:9242) (Cell Signalling Technology, Danvers, MA), p65(Cat:PA5‐16545), AKIP1(Cat:PA5‐106533), p‐Akt (Ser473)(Cat: OMA1‐03061), Akt(Cat:44‐609G), (Thermofisher, Massachusetts) were used during subsequent membrane incubation for 12 hours at 4°C. Following rinsing extensively with Tris‐buffered saline‐Tween 20, membranes were blotted with secondary antibodies conjugated to horseradish peroxidase (HRP) (Bio‐Rad) for 1 hour, and chemiluminescence kit was used to detect the signal intensity, which was quantified with densitometry using NIH ImageJ software (ImageJ, RRID: SCR_003070) and a Xerox scanner.

### 2.10 Luciferase reporter assay

Cotransfection of HeLa cells with pGL4.32 vectors and pRL4‐TK Renilla luciferase constructs (Promega, Madison, WI) was performed using Lipofectamine 2000, as previously described.^13^ The cells were further transfected 24 hours later with empty vectors (EV) or AKIP1‐expressed plasmid. Ly294002 (40 μM) or Bay117082 (15 μM) was used for treatment of AKIP1‐transfected cells for 4 hours, and 24 hours later, dual luciferase reporter assay was conducted with EV‐transfected cells that were pre‐treated with TNF‐α (40 ng/mL) as positive control. 24 hours after transfection, reporter assay was performed in triplicate, and all of the values of firefly luciferase activity were normalized based on the values of Renilla luciferase.

### 2.11 Wound healing assay

In total, cells (3 × 10^5^/well) were incubated for 24 hours, and subsequently, a wound was made in cells in a medium containing 1% bovine serum albumin. In six regions, the course of cell migration was imaged (after identifying each of the wounded zones), immediately after wounding and 48 hours thereafter (0‐48 hours) mounted on the inverted microscope (Nikon TMS‐F, 301655). Cell migration was illustrated by the rate of migration: (original width of scratch − new width of scratch)/original width of scratch × 100%.

### 2.12 Cell invasion

Assay of cell invasion was performed as previously described.^14^ In brief, cells were placed on an insert of polycarbonate membrane located inside a transwell (Corning, New York, NY) coated with fibronectin. We rinsed the insert and, after 8 hours of culture, cells on the upper chamber were removed while the remaining cells were fixed in methanol and stained with crystal violet. The number of cells with a positive signal was assessed under microscope in five fields (×100) to calculate the average. All assays were carried out three times.

### 2.13 Immunohistochemistry (IHC)

The paraffin sections of the specimens (4 μm thick), were treated with 100% xylene and hydrated ethanol for gradient dehydration in accordance with the standard protocol. The endogenous activities of peroxidase and non‐specific antigen were inhibited by a blocking reagent, followed by incubation with antibodies at 4°C and vigorous rinsing. The slide was incubated with rabbit anti‐goat antibody labelled by biotin performed at room temperature for 15 minutes before subsequent incubation. With a semi‐quantitative grading system, IHC was scored according to following criteria by two blinded pathologists independently^15^: 0 point for section containing <5% positive cells, 1 point for 5% to 30%, 2 points for 31% to 70% and 3 points for ≥71%.

### 2.14 Statistical analysis

Mean ± SD was used for data expression. Comparisons were done using One‐way ANOVA or a Student's *t* test and Tukey's multiple comparison. ImageJ were used for statistical analysis of the wound healing array. All probability values were two‐sided. SPSS 22.0 software (RRID: SCR_002865) was employed for statistical analysis. *P* < .05 suggested that difference had statistical significance.

## 3 RESULTS

### 3.1 Increased AKIP1 expression in CC cells

**FIGURE 1:**
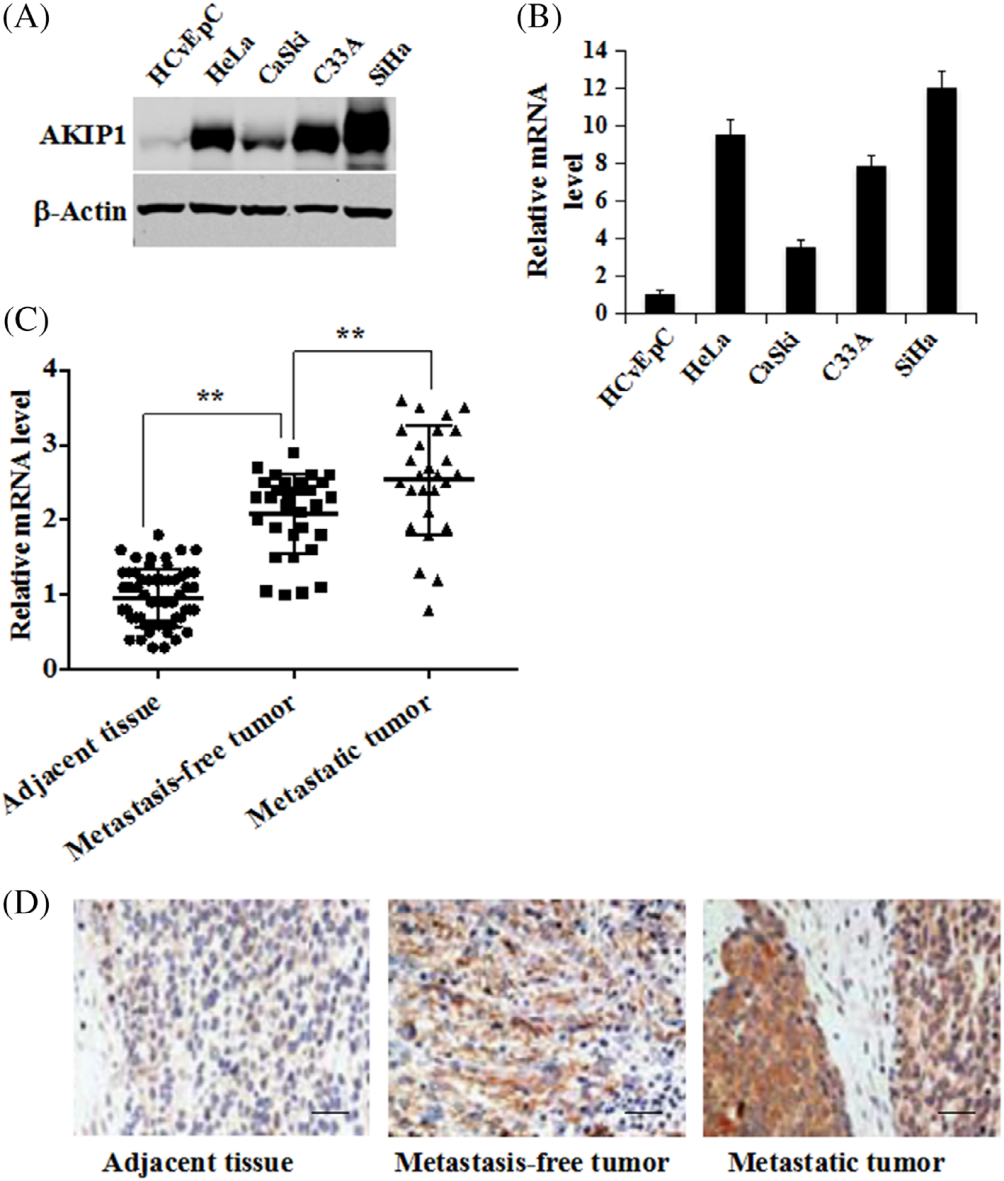
Upregulated AKIP1 expression in CC cells and tissues. The expression of (A) AKIP1 protein and (B) mRNA in HCvEpC, HeLa, CaSki, C33A, and SiHa cells. The expression of AKIP1 in 57 pairs of cervical cancer (CC) and adjacent noncancerous cervical tissues (31 pairs collected from lymph node and distant metastases patients, another 26 pairs from metastasis‐free patients) was determined using (C) qRT–PCR measurements and (D) immuno‐histochemical staining. Mean ± SD is used for the description of data in three independent measurements. ** indicates *P* < .01, as compared to the control. The scale bar is 100 μm

Expression of both AKIP1 protein and mRNA was markedly increased in CaSki, HeLa, SiHa and C33A cell lines in comparison with the normal human cervical epithelial cells (HCvEpC) (Figure 1A and B).

### 3.2 AKIP1 upregulation correlates with CC progression

We conducted qRT‐PCR analysis on 57 pairs of CC specimens to examine AKIP1 expression levels in CC, with adjacent tissue specimens as control. Figure 1C indicates that the mRNA expression levels of AKIP1 are significantly higher in primary CC tissues than in the matched adjacent normal tissues (*P* < 0.001). mRNA levels of AKIP1 in metastatic tumours were also significantly higher than in metastasis‐free tumours (*P* < .001). Figure 1D shows that AKIP1 was highly expressed in CC tissues but rarely in normal tissues. Moreover, CC specimens from patients with distant metastases demonstrated higher AKIP1 expression than metastasis‐free patients (Figure 1D).

### 3.3 Overexpression of AKIP1 promotes CC cell proliferation in vitro

**FIGURE 2:**
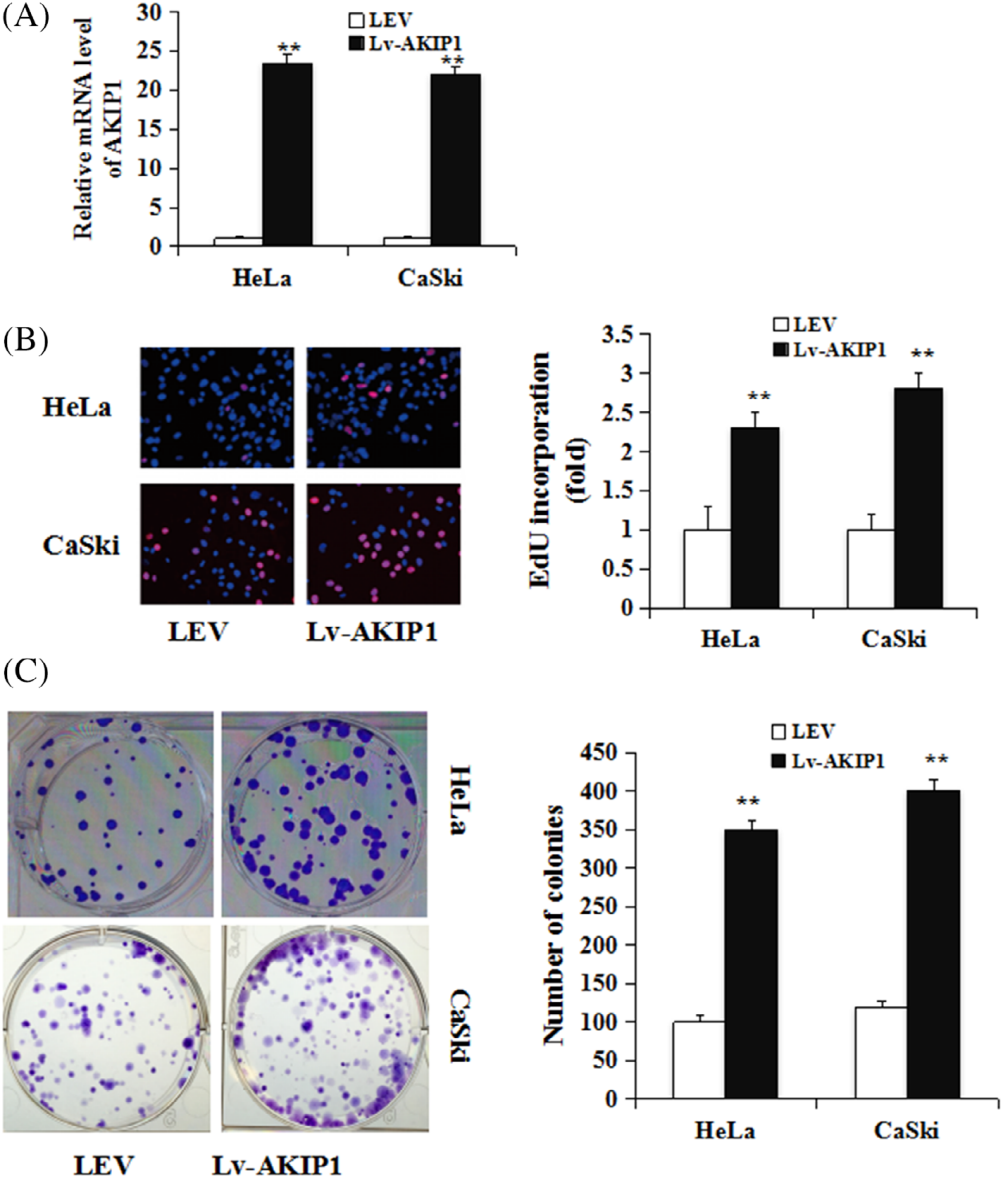
Overexpression of AKIP1 promotes CC cell growth. A, Stably transfected lentiviral vector (Lv‐AKIP1) overexpressing the AKIP1 gene and LEV in CC HeLa cells and CaSki cells. qRT‐PCR was used for the determination of AKIP1 mRNA expression. B, EdU incorporation assay, Left, representative image of EdU staining; Right quantification of EdU positive cells. C, colony formation assay were used to determine the influence of Lv‐AKIP1 or LEV on HeLa cells and CaSki cells. Mean ± SD is used for the description of data from three independent measurements. **indicates *P* < .01, as compared to the control

We stably transfected a lentiviral vector (Lv‐AKIP1) overexpressing AKIP1 and an empty control vector into human CC HeLa and CaSki cells to investigate its biological functions in CC. qRT‐PCR measurement showed an increase of more than 20‐fold in the AKIP1 expression of Lv‐AKIP1‐HeLa cells and Lv‐AKIP1‐CaSki cells (Figure 2A) compared with the control leti‐virus empty (LEV) group. Subsequently, in‐vitro growth of CC cells was determined. As shown in Figure 2B, the proliferation of Lv‐AKIP1‐HeLa and Lv‐AKIP1‐CaSki cells was greatly enhanced by Lv‐AKIP1 compared with LEV. In addition, colony formation assays revealed that ectopic AKIP1 expression increased CC growth (*P* < 0.01, Figure 2C). These observations indicate that AKIP1 in vitro promotes the proliferation of CC cells.

### 3.4 AKIP1 regulates EMT in CC cells and enhances the ability of migration and invasion

**FIGURE 3:**
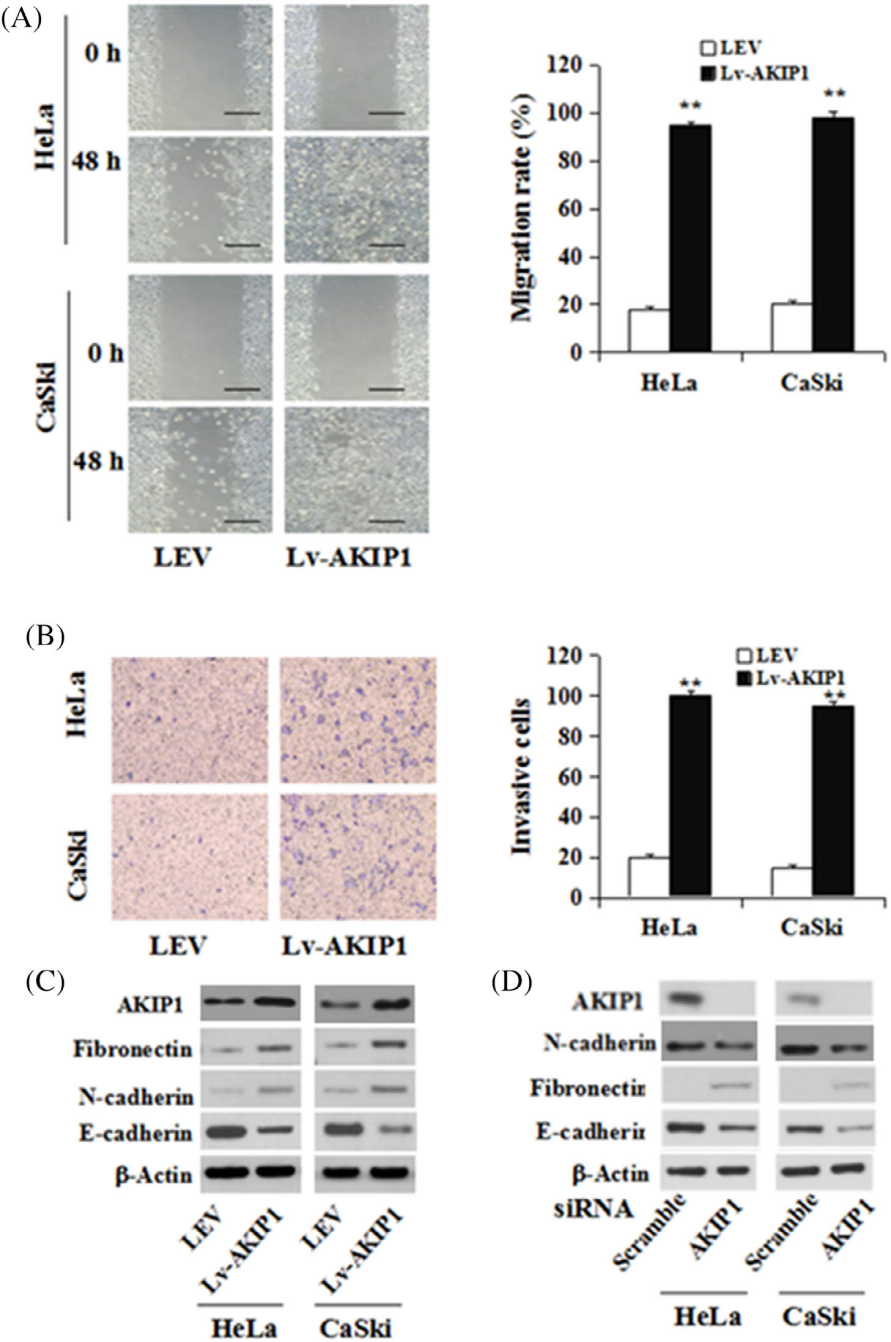
AKIP1 affects CC cell motility and invasion in vitro. A, A scratch wound healing assay and B, cell invasion assay were performed on Lv‐AKIP1‐HeLa, Lv‐AKIP1‐CaSki, and LEV cells. Mobility and invasion rate histograms of each group are shown in the left panel. C, Immunoblotting of fibronectin, N‐cadherin, and E‐cadherin in Lv‐AKIP1‐HeLa and Lv‐AKIP1‐CaSki cells. D, Immunoblotting of fibronectin, AKIP1, and E‐cadherin in HeLa and CaSki cells transfecting with siRNA against AKIP1 or scramble. Mean ± SE is used for the description of data from three independent measurements. ** indicates *P* < .01, as compared to the control

In this study, it was postulated that AKIP1 may act in CC cell metastasis, and how AKIP1 affects metastasis was investigated. Compared to LEV cells, Lv‐AKIP1‐HeLa and Lv‐AKIP1‐CaSki CC cell lines manifested a significant increase in migration ability (*P* < 0.01, Figure 3A). Culture of LEV and Lv‐AKIP1 cells was performed in a Boyden chamber for 8 hours; later, invasion of Lv‐AKIP1‐HeLa and Lv‐AKIP1‐CaSki cells throughout the matrigel was observed, and there was a significant increase invasion compared to the controls (both *P* < 0.01; Figure 3B). Moreover, a mesenchymal phenotype was indicated by a decrease in E‐cadherin and an increase in fibronectin (Figure 3C). Oppositely, suppression of expression AKIP1 with siRNA significantly enhanced E‐cadherin but abrogated fibronectin in both Hela and CaSki cells (Figure 3D). These results suggested that cell migration, invasion and EMT are promoted by AKIP1in CC.

### 3.5 AKIP1 promotes EMT through NF‐κB signalling

**FIGURE 4:**
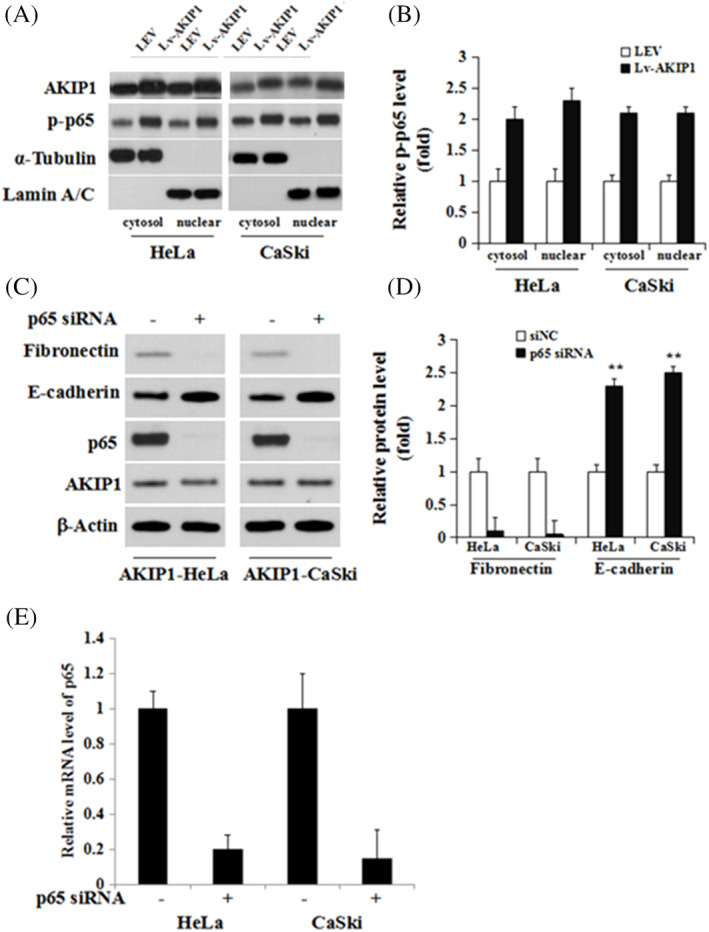
NF‐κB is activated and participates in the AKIP1 mediated induction of the EMT. A, The cytoplasmic and nuclear proteins in Lv‐AKIP1‐HeLa, Lv‐AKIP1‐CaSki, and control LEV cells were extracted. Levels of indicated proteins were measured using western blot. B, Measurement of protein signal intensities using ImageJ software. C, AKIP1‐HeLa and Lv‐AKIP1‐CaSki cells were transfected with siRNA‐negative control (siNC) or p65 siRNA transiently for 72 hours to knock down p65. The expression levels of AKIP1, p65, E‐cadherin, and fibronectin proteins were detected using western blot analysis. D, Measurement of protein signal intensities was done using ImageJ software. E, Knockdown of p65 using siRNA in Hela and CaSki cells was confirmed using RT‐PCR. Mean ± SD is used for the description of data from three independent measurements. ** indicates *P* < .01, as compared to the control

As NF‐κB can modulate the EMT phenotype in gastric cancer,^16^ it is thought to be related to the AKIP1‐induced EMT. Therefore, Western blot was performed to detect the expression of p65 protein in CC cell lines with AKIP1 overexpression. Levels of p‐p65, the activated nuclear form, were significantly higher in Lv‐AKIP1‐HeLa and Lv‐AKIP1‐CaSki cells than in control LEV cells (Figures 4A and B). p65 was silenced in AKIP1‐overexpressing HeLa cells using siRNA (Figure 4E), and we found that the AKIP1‐mediated variations in E‐cadherin and fibronectin expression were reversed by p65 knockdown (Figures 4C and D).

### 3.6 AKIP1 activates NF‐κB signalling

**FIGURE 5:**
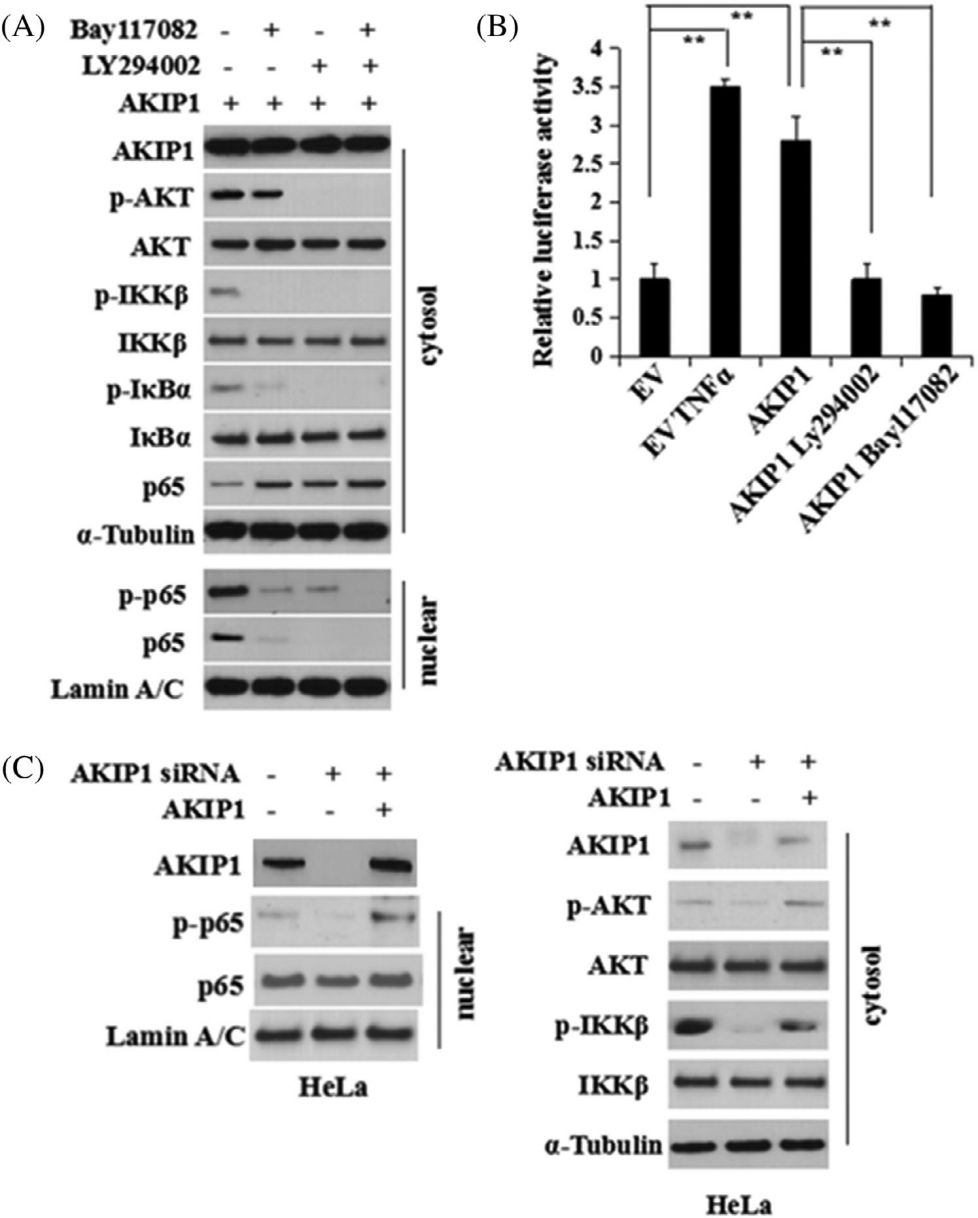
AKIP1 mediates NF‐κB regulation via the Akt/IKKβ pathway during the EMT. A, Serum‐starved HeLa cells were pretreated with PI3K inhibitor Ly294002 (40 μM) or IKK inhibitor Bay117082 (15 μM) for 4 hours, followed by stimulation with AKIP1 protein (80 ng/mL) for 4 hours. Cell lysates were harvested and the levels of indicated proteins were measured using western blot analysis. B, pGL4.32 (luc2NF‐κB‐RE/Hygro) vector and pRL4‐TK Renilla luciferase constructs were used for co‐transfection of HeLa cells. The cells were further transfected 24 hours later with empty vector (EV) or AKIP1 expression plasmid. Some of the AKIP1‐transfected cells were pretreated with Ly294002 (40 μM) or Bay117082 (15 μM) for 4 hours. The dual luciferase reporter assay was performed 24 hours following transfection of AKIP1. EV‐transfected cells treated with TNFα (40 ng/mL) for 1 hour after transfection were used as the positive control. ** indicates *P* < .01. C, The HeLa cells were transfected with AKIP1 siRNA to knock down AKIP1. After 72 hours transfection, AKIP1 protein (80 ng/mL) was used to stimulate HeLa cells for 4 hours and the cell lysates were analysed using western blot

Based on the fact that activation of NF‐κB in cells transformed by RAS is mediated via PI3K/Akt pathway,^17^ it was proposed that NF‐κB is activated by AKIP1 in CC through PI3K/Akt pathway. Activation of p65 by IκB kinase beta (IKKβ) results in nuclear translocation of p65.^18^ The results show that AKIP1 failed to activate p65 when either the PI3K/Akt or the IKK pathway was blocked (Figure 5A). Luciferase reporter assay showed that in transient AKIP1‐transfected HeLa cells, the transcription of NF‐κB was higher than that in cells transfected by EV. (*P* < 0.001, Figure 5B). After treatment of PI3K or IKK inhibitor, a decrease was identified in NF‐κB transcription (*P* < 0.001, Figure 5B). AKIP1 knockdown decreased the phosphorylation of Akt and IKKβ and the nuclear translocation of p65 and p‐p65; these changes were reversed after the cells were treated with AKIP1 (Figure 5C).

**FIGURE 6:**
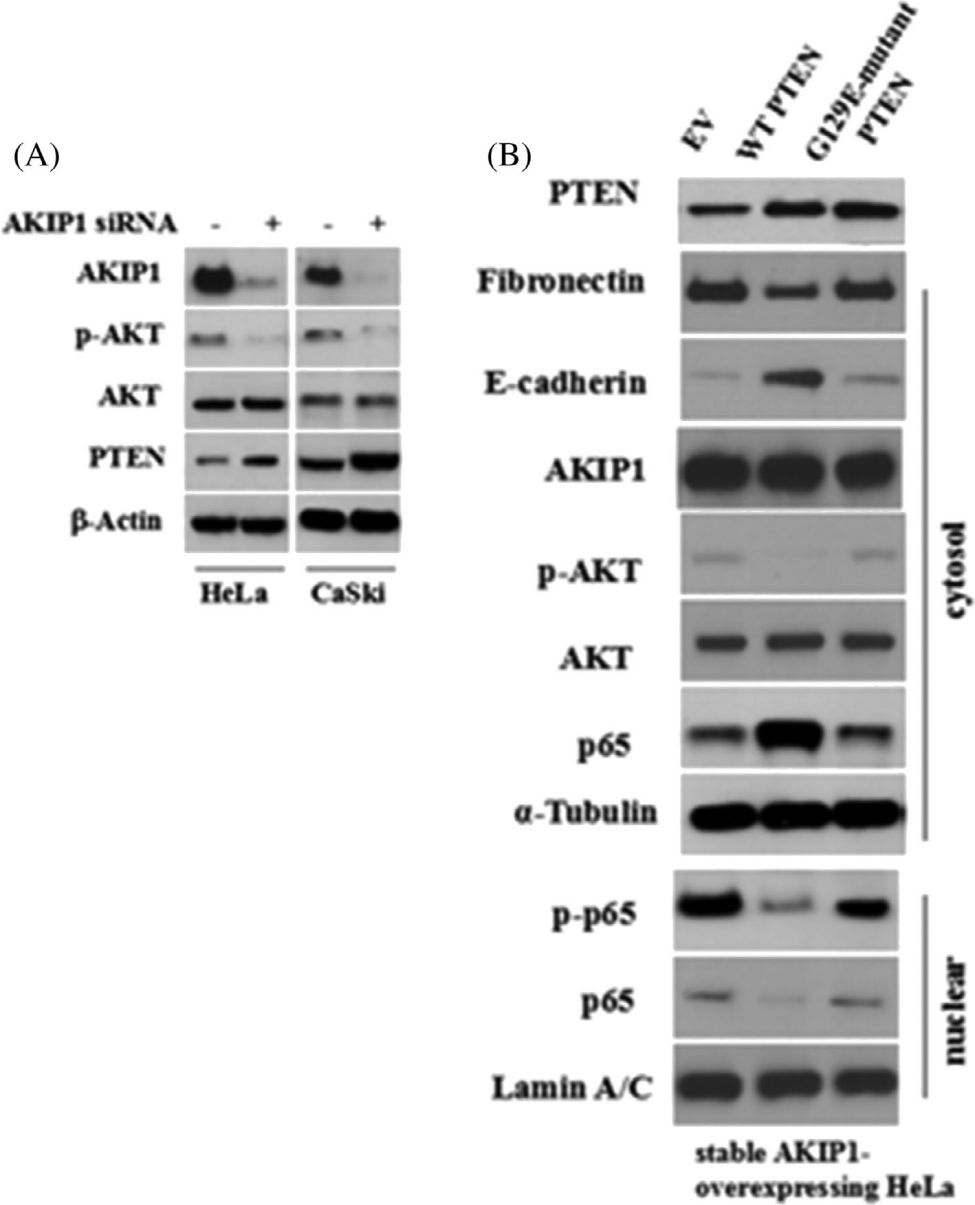
AKIP1 Suppresses PTEN during the EMT. A, HeLa and CaSki cervical cancer cells were transfected with a control siRNA (siNC) or AKIP1 siRNA. The cell lysates were collected and the protein expression of PTEN, AKIP1, p‐Akt, total Akt, and β‐Actin was detected using western blot. B, Stable AKIP1‐expressing cells were transfected with EV, wild‐type PTEN, or G129E‐mutant PTEN plasmids. At 48 hours after transfection, cell lysates were harvested after cell fractionation. Levels of indicated proteins were determined using western blot analysis

PTEN acts as tumour suppressor in CC.^19^ Control siRNA or AKIP1 siRNA was transfected into HeLa and CaSki cells. Figure 6A shows that in transfected cells, changes to PTEN expression were reversed by AKIP1. Furthermore, wild‐type or mutant PTEN were transfected into HeLa cells that could overexpress AKIP1 stably, and transfection with wild‐type PTEN suppressed the nuclear translocation of p65 and restored E‐cadherin expression in these cells, whereas transfection with mutant PTEN did not (Figure 6B).

## 4 DISCUSSION

In this study, in patients with CC, the high expression of AKIP1 indicates a risk of metastasis, and it is potentially a prognostic factor,^20^ suggesting that improving reregulation of AKIP1 can assist clinicians to predict the risk of metastasis in lymph nodes. Elevated expression of AKIP1 protein is involved in pelvic lymph node metastasis. Moreover, upregulated AKIP1 significantly affects the motility and invasion ability of CC cells, potentially through NF‐κB pathway‐regulated EMT. The results of this study suggest that AKIP1 may aid in the metastasis of CC. In addition, the expression profile of AKIP1 is an innovative predictor of pelvic lymph node metastasis, which is also a risk factor for CC patients. Our results indicate that AKIP1, a potential regulator in EMT axis, promotes EMT and metastasis in CC by activating NF‐κB signalling, which might be achieved through PI3K/Akt/IKKβ pathway.

There are limited number of studies on the biochemical and biological functions of AKIP1. It is considered a regulator cardiac stress adaptation, as overexpression of AKIP1 in the cardiac tissues, shields the heart from ischemia/reperfusion and refined heart function. Besides, AKIP1 expression is elevated in both tumour cells and in the plasma in many types of cancer, including CC. It is also considered an oncogenic protein is involved in tumorigenesis and invasiveness by regulating protein kinase A (PKA) and nuclear factor kappa‐B (NF‐κB) signalling in breast cancer. However, the underlying mechanism in CC is mostly unclear.

EMT activation may enhance cancer cell motility and metastasis.^21^ This study confirmed that the overexpression of AKIP1 could induce EMT in CC cells, consistent with the pro‐invasive function of AKIP1 in CC. Western blot analysis also demonstrated a reduction in the level of epithelial E‐cadherin. However, the level of mesenchymal fibronectin and N‐cadherin expression were increased in CC cells overexpressing AKIP1. AKIP1 is a positive regulator of EMT in CC cells, suggesting that the EMT pathway could become a target to prevent tumour metastasis.^22^ The metastasis of various human cancer cells is promoted by EMT^23^ and is regulated by a number of signalling pathways, including the NF‐κB pathway.^24^ Consistent with previous reports,^24^, ^25^ this study found that the metastasis of cancer cells was promoted by AKIP1 via the EMT, which was regulated by the NF‐κB pathway. However, the detailed molecular mechanism underlying AKIP1 interaction with NF‐κB pathways during the induction of the EMT, which facilitates the metastasis of CC cells, requires further investigation in future studies. E‐cadherin and fibronectin are the cancer adhesion molecules whose loss is required for the acquisition of an invasive phenotype. p65 knock down in CC cells down‐regulates endogenous E‐cadherin and fibronectin expression, maybe overexpression of p65 can increase E‐cadherin and mRNA and protein levels thereby increase CC invasiveness in vitro.

AKIP1 is not only involved in CC progression, but also is critical to NF‐κB signalling. It was shown that activation of NF‐κB is involved in AKIP1‐induced EMT in CC.^26^ NF‐κB signalling is indispensable for EMT.^27^, ^28^ This study revealed that the activation of p‐p65 was increased notably in AKIP1‐overexpressing CC cells. After p65 knockdown, the AKIP1‐mediated changes in the expression of E‐cadherin and fibronectin was reversed. Therefore, AKIP1 failed to promote EMT when NF‐κB was blocked, suggesting the involvement of NF‐κB signalling pathway in AKIP1‐induced EMT. PTEN is considered as a tumour suppressor in CC with minimal relevance.^19^ Our study is the first to find that there is an inverse regulatory and functional relationship between AKIP1 and PTEN in CC. Through counteracting AKIP1 function, PTEN overexpression repressed NF‐κB activation and inhibited the EMT. Together, these findings indicate that AKIP1 may activate the NF‐κB signalling through the PI3K/Akt/IKKβ pathway by inhibiting PTEN during the EMT. However, there are some limitations to this study. Recent studies showed involvement of mTOR and MDM2 in EMT,^29^, ^30^ this study did not rule out their influence with AKIP1 which should be confirmed further with novelty methods.

AKIP1 expression in the samples of CC patients and in in vitro tumour cell lines provides evidence that expression of AKIP1 in CC contributes to cancer progression. Our findings indicate that AKIP1 promotes the EMT and metastasis in CC by activating NF‐κB signalling through the PI3K/Akt/IKKβ pathway, suggesting AKIP1 as a pivotal regulator of an EMT axis in CC. AKIP1 could be implicated as a potential therapeutic target as well as a valuable biomarker for evaluating prognosis for patients with CC.

## CONFLICT OF INTEREST

The author declared no potential conflicts of interest with respect to the research, authorship, and/or publication of this article.

## Data Availability

The datasets used and/or analyzed during the current study are available from the corresponding author on reasonable request.
